# Setting the balance of care for older adults at risk of hospitalization and delayed discharge: A mixed-methods research protocol

**DOI:** 10.1371/journal.pone.0315918

**Published:** 2024-12-17

**Authors:** Kerry Kuluski, Danielle Jacobson, Somayeh Ghazalbash, Junhee Baek, Laura Rosella, Elizabeth Mansfield, Abhimanyu Sud, Terence Tang, Sara J. T. Guilcher, Manaf Zargoush

**Affiliations:** 1 Institute for Better Health, Trillium Health Partners, Mississauga, Ontario, Canada; 2 Institute of Health Policy, Management, and Evaluation, University of Toronto, Toronto, Ontario, Canada; 3 Health Policy and Management Area, DeGroote School of Business, McMaster University, Hamilton, Ontario, Canada; 4 Dalla Lana School of Public Health, University of Toronto, Toronto, Ontario, Canada; 5 Institute for Clinical Evaluative Sciences (ICES), University of Toronto, Toronto, Ontario, Canada; 6 Centre for AI Research and Education in Medicine, Department of Laboratory Medicine and Pathobiology, Temerty Faculty of Medicine, University of Toronto, Toronto, Ontario, Canada; 7 Department of Occupational Science and Occupational Therapy, University of Toronto, Toronto, Ontario, Canada; 8 Humber River Health, North York, Ontario, Canada; 9 Department of Family and Community Medicine, University of Toronto, Toronto, Ontario, Canada; 10 Department of Medicine, University of Toronto, 6 Queen’s Park Crescent West, Toronto, Ontario, Canada; 11 Department of Physical Therapy, Temerty Faculty of Medicine, University of Toronto, Toronto, Ontario, Canada; 12 Leslie Dan Faculty of Pharmacy, University of Toronto, Toronto, Ontario, Canada; PLoS ONE, UNITED STATES OF AMERICA

## Abstract

**Introduction:**

Delayed hospital discharge is a persistent care quality issue experienced across health systems worldwide and remains a priority area to be addressed in Canada. Often associated with a decrease in services while waiting to leave the hospital, delayed discharge from hospital can lead to increased frailty, physical and cognitive decline, and caregiver burnout. Optimizing availability of and timely access to community-based health and social care are avenues that could reduce initial admissions to the hospital and length of hospital stay, and facilitate hospital discharges.

**Methods:**

This research will explore the ways in which community resources could be leveraged to potentially avoid hospitalization and delayed hospital discharge for older adults using sequential mixed-methods including co-design. To better understand the characteristics and needs of older adults, the research team will first identify sub-populations of older adults (65 years old or older) at risk of hospitalization and delayed discharge using comprehensive, longitudinal administrative health data. From these health data, risk profiles and personas will be created and then shared with key partners (e.g., older adults, caregivers, healthcare providers, healthcare decision-makers), who will be engaged to identify, leverage, and create targeted care solutions. The barriers and facilitators to the implementation of these care solutions will then be explored.

**Discussion:**

Delayed hospital discharge has been a critical care quality issue across Canada for decades. The current research will provide health system leaders with an approach to better allocate services to older adults in order to avoid delayed hospital discharge and identify gaps in health and social care resources based on the characteristics, needs, and preferences of older adults, their caregivers, and providers.

## Introduction

Demands for hospital beds are high in the Canadian healthcare system [1]. There are over 3 million acute care hospitalizations in Canada each year [2], and approximately 20% of patients end up “getting stuck” in hospital following the completion of their acute medical treatment [3]. This means that while treatment is completed, non-medical considerations (often social in nature) prevent successful discharge. In Canada, this phenomenon is referred to as Alternate Level of Care (ALC) [4, 5] and more commonly referred to as delayed hospital discharge in other health systems [6]. Finding solutions to address delayed hospital discharge has been a major priority in Canada for several decades [5, 7], but the problem persists.

To address these pressures in the healthcare system, it is critical to look at how hospitalizations and delays in discharge can be avoided, especially via “continuous and coordinated care practices” upstream (p.1) [8]. Most of the research to date on delayed discharge has focused on the characteristics of patients who have delayed hospital discharge status (e.g., majority 75 years old or older, without a live-in caregiver, has co-morbidities, lower socioeconomic status) as well as strategies to improve patient flow *once they are already in hospital*. In McGilton et al.’s scoping review on transitional care programs for individuals at risk of delayed discharge, the authors highlighted the importance of improving acute or community care practices upstream rather than continuing to reactively address the individual health consequences “that occurred in hospitals” (p. 15) [9]. There is a significant gap in research focusing on the prevention of delayed hospital discharge, such as earlier access to community resources (defined as formal or informal health and social support, whether part of or independent from services offered within the healthcare system, delivered to individuals who reside outside of the hospital in a home or other community-based residence) among people at risk. Also, in previous studies, the perspectives and lived experiences of patients and caregivers are limited [9–11]. As COVID-19 and its legacy impacts have placed additional strain on hospital resources, it is even more important to explore under what conditions and for whom, hospitalization and subsequent delayed hospital discharge designation can be avoided or minimized.

Part of facilitating a better understanding of how to proactively address delayed hospital discharge is the Balance of Care approach [12, 13]. The Balance of Care was established in the United Kingdom (UK) and applied in Ontario, Canada, as a health system planning tool to optimally distribute existing resources in order to better care for aging populations [14, 15], with some studies conducted from the standpoint of aging populations with mental health conditions [16, 17], as well as senior management and frontline providers [18]. In previous research in Ontario, the Balance of Care approach has been used to address long waitlists for long-term care [19] and access to services for aging individuals in rural settings [12, 20].

The study aims to investigate how to prevent delayed hospital discharge by examining what health and social resources are needed in the community. Finding the right “balance of care” between hospital and community is required to ensure that older adults are empowered to be in the place of care that makes the most sense for their needs and preferences.

## Materials and methods

### Study objectives, design, and research questions

This is a 4-phase explanatory sequential mixed-method study. Leveraging the expertise of an interdisciplinary team in machine learning (ML), co-design, community engagement, mixed-methods, and delayed hospital discharge, the authors will address the following objectives:

1. Better understand the characteristics and needs of older adults (65 years of age and older) at risk of hospitalizations that lead to a delayed discharge.
2. Understand how community resources could be leveraged to avoid hospitalization of older adults.
3. Generate an approach to better allocate existing resources, understand the barriers and enablers in doing so, and disseminate this knowledge within communities.

The Balance of Care approach informs this research as the research team will build more in-depth knowledge of the characteristics of older adults at risk of hospitalization and co-design care packages to better understand the balance of care required to better support this population in the community and avoid hospitalization and ALC designation.

The research question for the quantitative phase is: How can supervised machine learning algorithms be developed and validated to accurately predict the risk of delayed-discharge hospitalization and identify the characteristics of older adults with such risk? The research questions for the qualitative phase are: What would community care look like for older adults at risk of being designated with delayed discharge status? What would hospital care look like for people who are at risk of being designated with delayed discharge status? What are the barriers and facilitators to providing community care as well as optimal hospital care for older adults at risk of hospitalization with a delayed discharge status?

### Ethics

The quantitative part of this research including data and related analysis are housed at and led by researchers (MZ and SJTG) at a university for which there are separate ethical provisions approved by the Hamilton Integrated Research Ethics Board (HiREB; REB # 5472) in Ontario.

The qualitative portion of this research has been approved by the Research Ethics Boards at Trillium Health Partners (THP; ID#1196), Royal Victoria Regional Health Centre (RVRHC; REB # R23-039) and University of Toronto (REB #45827). Humber River Hospital (HRH) has recently established their Research Ethics Board; the authors are preparing a research ethics application for submission.

### Positionality

The research team comprises researchers who share a common goal–to improve the quality of life for aging populations in Ontario by contributing to strengthened support to avoid or minimize hospitalization, where possible. For some team members, this goal is motivated by individual lived experiences supporting people to navigate the healthcare system, while for other team members, this goal is motivated by a passion for health systems improvement. Entering into the research, the research team acknowledges each individual’s unique lived experiences and perspectives, which, alongside a recognized gap in care for aging populations, have directed the formation of this project.

Who each individual on the research team is as a researcher, including their experiences and backgrounds, has an impact on the work [21–23]. The research team holds various positions as researchers, ranging from scientists, research associates, associate professors, as well as clinicians. Team members hold diverse training in public health, psychology, social work, medicine, and physical therapy, and specialized expertise in aging populations, women’s health, quantitative, qualitative, mixed methods, and the balance of care approach. The authors acknowledge the privilege entwined in their positions as researchers in university and hospital settings, with the potential to make positive changes for aging populations and providers who care for this population. This contributes to the power dynamic between themselves as researchers and potential aging participants who may be experiencing instability.

As team members enter into the research, they will continue to engage in reflexive conversations about their multi-faceted identities and the ways in which who they are as researchers in the world have an impact on data collection, analysis, and dissemination [21]. These conversations will be supported by A) the Social Identity Map [24], a tool that helps researchers to reflect on their positionality and how it influences the research process, and B) the coin model of privilege and critical allyship [25], which will help team members to reflect on their approach to solidarity in addressing healthcare system inequities.

### Study setting

This study will comprise three separate sites in Southern Ontario, Canada (THP, HRH, and RVRHC). THP is in Mississauga (the 6th largest city in Canada), located in the Peel region of Ontario, and serves Mississauga and surrounding areas, including West Toronto. Mississauga is made up of 1.4 million residents and is described as one of the most diverse regions in Canada [26]. Just over half of Peel’s population are immigrants, and approximately half report low income. Located in one of the most diverse cities in the world, HRH is a hospital in Toronto, serving approximately 850,000 residents in the surrounding areas, including Northwest Toronto, Etobicoke, York Region, and Rexdale. Northwest Toronto and the surrounding area have one of the most racialized populations in Canada, and the community also has a high proportion of seniors aged 80 and above. RVRHC is located in Barrie, Ontario, in the Simcoe Muskoka Region, with a population of just under 600,000 [27]. Simcoe Muskoka has a rapidly aging population, for which the rate of preventable mortality is 9.2 times higher than non-aging populations in the same region. This aging population (65 years of age and older) comprises 20.8 percent of the overall population [27, 28].

These three sites, THP, HRH, and RVRHC, were selected to ensure diversity in patients, diversity in geographic location, and to capture the varied experiences of potentially marginalized groups; both the City of Toronto and Peel region where THP is located have a higher concentration of immigrant and visible minorities [29, 30].

## Materials, methods, and data analysis: Quantitative investigation

### Population

For the quantitative investigation, secondary administrative health data will be used, which will include approximately 1.8 million patients with 12 million observations. These are patients 65 years and older who were admitted to an acute care facility. Data will be excluded from patients less than 65 years of age, from those who are non-Ontario residents, and if there is missing or invalid data.

### Data sources

Secondary administrative health data will be used from data housed at the Institute for Clinical Evaluative Sciences (ICES) in Ontario, Canada. ICES is an independent, non-profit research institute funded by an annual grant from the Ontario Ministry of Health and the Ministry of Long-Term Care. As a prescribed entity under Ontario’s privacy legislation, ICES is authorized to collect and use healthcare data for the purposes of health system analysis, evaluation, and decision support. The use of the data in this project is authorized under section 45 of Ontario’s Personal Health Information Protection Act (PHIPA) and is approved by the ICES privacy office; as such, it does not require further review by a research ethics board. Secure access to these data is governed by policies and procedures approved by the Information and Privacy Commissioner of Ontario. These datasets are linked using unique encoded identifiers and analyzed at ICES.

### Creating risk profiles: Quantitative data and analysis

To create robust risk profiles for patients at risk of delayed discharge, an analysis will be conducted using advanced ML techniques on extensive longitudinal data from ICES spanning approximately two decades (2004–2022). The Discharge Abstract Database (DAD) provides detailed records of patients’ acute care services, encompassing clinical information such as patient comorbidities and interventions coded according to ICD10, administrative data (e.g., length of stay), and the outcome of interest (i.e., ALC designation). To supplement this dataset, patient demographic and socioeconomic characteristics will be sourced from the Registered Persons Database (RPDB), which monitors individuals registered for healthcare services in Canada [31].

Following the common approach in ML, The first step is to divide the data into training (90%) and test (10%) sets. Given the large size of the datasets, the research team opts for a 90–10 split to have a balance between having sufficient data for training the model while still reserving enough data for reliable performance evaluation. The training data will be used to develop the ML-based predictive model. The test data, which the model does not utilize for training, will be used to evaluate predictive performance and reliability. This evaluation will be done using various metrics such as accuracy, area under the curve (AUC), sensitivity, specificity, and calibration.

After ensuring the model’s accuracy, and to create a transparent and easy-to understand model, explainable ML techniques will be employed, such as Shapley Additive Explanation (SHAP) values [32] and Breakdown values [33]. These techniques will help the research team to quantify the impact of each risk factor on the predicted risk of delayed discharge for each patient, enabling the research team to provide a personalized and coherent explanation for the projected risk. Subsequently, patients will be categorized into different risk levels (e.g., low, medium, and high) based on their risk of delayed discharge. For each risk group, critical risk factors will be identified based on the consensus on the Shapley and Breakdown values, which contribute to the group’s risk level. This analysis enables an understanding of each group’s risk profile, pinpointing key factors for personalizing community care interventions at different levels of delayed discharge risk.

The data-driven analytics consists of four main steps: 1) data preparation, 2) development of predictive models, 3) feature identification, and 4) development of risk profiles. In the data preparation phase, look-ahead windows will be constructed for 90, 180, and 365 days to predict whether patients will be designated a delayed discharge within those time frames after their admission. Additionally, a 2-year lookback window will be created to gain a comprehensive understanding of the patient’s health history prior to the visit.

Second, several supervised ML-based predictive models will be developed to accurately predict the likelihood of a patient encountering delayed discharge following hospitalization. Specifically, Gradient Boosting Decision Tree algorithms (GBDT) will be used [34]. The research team has previously successfully applied these algorithms to health administrative data and found them to perform well given the data and this type of classification task [35–38]. For benchmarking purposes, logistic regression will also be used as the traditional prediction algorithm in healthcare.

Third, the important features chosen in step two will be identified and ranked according to their importance based on the consensus of SHAP and Breakdown values. The risk factors that contribute most to the model’s predictions will then be pinpointed [39]. These values will indicate how much each risk factor influenced the model’s prediction for a specific individual compared to the average prediction for all individuals. The top 10 most agreed-upon features will be examined using measures such as Kappa statistics [40, 41].

Fourth, the delayed discharge risk profiles will be identified based on the pinpointed features in step three. To this end, three delayed discharge risk groups will be targeted: low-risk (L), medium-risk (M), and high-risk (H). The choice of "three" subgroups is made to balance the trade-off between information gain through increased granularity (the higher the number of subgroups, the better) and computational difficulty (the lower the number of subgroups, the better). This will be done using two different approaches: A) The three risk groups will be equally distributed among the identified risk range (i.e., splitting the quantified risks into three equal ranges based on the formula (maximum risk–minimum risk)/3.; B) Using mathematical approaches to identify the best two thresholds *r*1 and *r*2 for creating three risk groups. As an illustrative example of the formula involved: the range of delayed discharge risk for group **L** = *minimum risk* − *r*_1_; the range of delayed discharge risk for group **M** = *r*_1_ − *r*_2_; and the range of delayed discharge risk for group **H** = *r*_2_ − *maximum risk*. The mathematical approach finds the best *r*_1_ and *r*_2_ that lead to maximal separation among L, M, and H groups. The qualitative investigation described in the next section will focus on the moderate- and high-risk groups to prioritize those most at risk.

These findings will inform the qualitative phase of the sequential mixed-method approach, which involves in-depth interviews and focus groups with patients, caregivers, healthcare providers, and healthcare decision-makers.

## Materials, methods, and data analysis: Qualitative investigation

In the design of the qualitative portion of this study, Tong, Sainsbury, and Craig’s COREQ checklist [42] has been consulted (see S1 Appendix). Items with a green check mark have been addressed in this manuscript. Items with a red “x” were unable to be addressed in this manuscript, given that the research has not yet begun.

### Advisory council

An advisory council will be recruited, which will include older adults and caregivers with delayed hospital discharge experience (including members of the existing Delayed Hospital Discharge Patient and Caregiver Advisory Council), clinicians, and researchers to seek their feedback on the research (e.g., focus group and interview guides, recruitment, data analysis, validation, study recommendations) [43]. Each advisor will be compensated with $40 CAD per hour as a token of appreciation for sharing their knowledge and expertise. Meetings are anticipated to occur two to three times or as needed.

The Central Region Access and Flow Table and the Tipping Point Task Force is comprised of organizational leaders from across the care continuum (homecare, community support services, para-medicine, hospital, and primary care) whose goal is to proactively address delayed discharge (squarely aligned with the goals of this project) and will also work in partnership with the research team.

### Participants

For the qualitative investigation, participants will include older adults at risk of (or who have experienced) a hospitalization that resulted in a delayed discharge (hereon referred to as an older adult), care partners (family and friends who provide care to patients), and care providers.

#### Older adults and caregivers

Older adult and caregiver participants who had previously experienced a hospitalization that resulted in a delayed discharge designation will be purposively recruited, as well as community-dwelling older adults who have experienced hospitalization in the past. The aim is to engage people that reflect each risk profile (e.g., across sex, gender, disease, and social characteristics, including racialized populations and residents with low income). The quantitative datasets on which the risk profiles will be based have limited data on social characteristics and determinants of health that may make someone vulnerable to health-system shortcomings (e.g., gender diversity, access to transportation, socio-economic resources). Therefore, the research team will aim to recruit a diverse sample for the qualitative investigation as well as probe for additional vulnerabilities during focus groups. Recruitment will be open, as well, to older adults who are community-dwelling and receiving services in the community and their caregivers who did not have hospitalization or delayed discharge designation but who may consider themselves frail and at risk of hospital care. It is also important to understand the experiences, needs, and perspectives of people with cognitive impairment who, too, are at risk of ALC. The recruitment strategy will therefore include this population and their caregivers. In the case that a prospective participant with cognitive impairment wishes to take part in the study, but does not have the cognitive capacity to participate in a focus group or interview independently, they will be required to be accompanied by a substitute decision-maker.

#### Healthcare providers and decision-makers

Healthcare providers and decision-makers will be purposively recruited for the qualitative investigation from the communities surrounding the three hospital sites. Recruitment will span across a range of health and social care organizations and professions (e.g., nurses, social workers, personal support workers, clinical managers, care coordinators, physicians) from across the care continuum (which may include the study hospitals, including their emergency departments, outpatient medical services, supportive housing/assisted living, retirement homes, homecare, community support services, immigrant services, etc.). Decision-makers will include managers and leaders across the health system who interact with or oversee care for delayed hospital discharge patients or frail seniors in the community. The research team will also purposively sample by sex and gender, given the role of gender in shaping experiences of human health resources [44]. In particular, gender has been reported as a “silent social stratifier”; male bias often permeates through the healthcare system, evidenced by protective equipment designed for male bodies, sexism in particular departments, and increased rates of stress and mental health risks for those in roles which women are most likely to fulfill (p. 13) [44].

## Flexible participation and co-design

The expectation is to conduct approximately ten to fifteen focus groups. Each focus group (not site specific) will have either older adult and caregiver participants or healthcare provider and decision maker participants. An interview option will be offered for any participant who prefers to participate one-on-one. Based on the estimated number of focus groups, the research team anticipates engaging 50 unique participants in total (15 older adults, 15 caregivers, and 20 healthcare providers and decision-makers) across the three sites, consistent with sample sizes that allow for sufficient information power [45]. The research team will also engage monthly with decision-makers in the region of study (Southern Ontario) to receive feedback on data findings and project steps, with the goal of greater uptake in practice upon study completion.

Participants will be able to participate in multiple focus groups across project stages to allow for continued learning and co-building. Co-design is a method that entails bringing different partner groups together to create a new (or refine an existing) process or product [46, 47]. In line with principles of engagement and co-design [43], sessions will be conducted to maximize comfort and opportunities for input for all participants.

### Recruitment

It is anticipated that recruitment will iteratively take place from approximately December 1^st^ 2024 to October 20^th^ 2025. For the qualitative investigation, recruitment flyers, email scripts, and social media materials will be co-designed with knowledge users and collaborators to distribute through their organizational mailing lists and post within their organizations. Social media (e.g., Twitter/X and Facebook) will also be used to promote recruitment for the qualitative portion of the study. Members of the research team will recommend hospital and community-based providers to reach out to and invite them to participate.

For older adults, families, and community members, a neutral third party (e.g., an administrator in home and community care organizations and hospital units) will be recruited to ask eligible patients, homecare clients, and caregivers if they would be willing to hear from a member of the research team to learn more about the study. Potential participants will also be given the contact information of a member of the research team so they can contact them directly to inquire about the study. As part of the onboarding discussion, prior to participating in the study, a member of the research team will explain to participants their personal and professional motivation for carrying out the research and will encourage participants to ask any questions they may have.

Participants will receive an honorarium in the form of a gift card as compensation for their contributions. Participants will be offered a $25 gift card for each one-on-one interview and a $50 gift card for each focus group they participate in. As of August 2024, recruitment and data collection has not yet begun for this research.

### Consent, confidentiality, and data management

A written-or-electronically-signed informed consent approach will be used by email. Verbal consent will be accepted and audio-recorded. Consent will be ongoing, and participants will be encouraged to ask questions throughout the study. If there are reasonable grounds to believe that the potential participant may lack the capacity to consent, a script/set of questions will be used to determine whether they understand the nature of participation, risks, benefits, and confidentiality (e.g., “In your own words, what will you do in the study? What are the risks and benefits to you?”). Consent will be sought from the appropriate substitute decision-maker(s) on behalf of older adults who lack the capacity to consent. After participants have consented (and prior to the focus groups), demographic data will be collected (e.g., age, sex at birth, gender identity, racial identity, a question related to financial strain).

All qualitative data will be analyzed exclusively at the host hospital study site (THP). Audio files will be placed on a secure server and the transcribed interviews will be password-protected. The transcripts will be read against the audio recordings to remove identifiable information and to check for accuracy. The audio files and transcripts will be kept for 10 years before being permanently deleted. Only researchers at the primary institute (THP) will have access to the data.

### Building risk profiles into personas

Based on the critical risk factors identified at each risk level from the ML model, risk profiles will be further developed into personas. In this context, personas are descriptions of the characteristics and needs of a segment of the population, which then gets written into a story and enriched with experiential information from participants; personas are therefore data and experience driven and are compiled from quantitative and qualitative data.

To develop the quantitative risk profiles into personas, first, the research team will write short vignettes or descriptions depicting the characteristics of the individuals in the sub-groups from the quantitative analysis. This first iteration of the personas will be presented to participants during qualitative focus groups and interviews for feedback. To support the development of the personas, the authors have created a worksheet, which will be shared during focus groups and interviews and will depict the quantitative variables and data (see S2 Appendix); the research team anticipates that this will include blank sections due to unavailable information from the quantitative data set. During focus groups and interviews, facilitators will prompt for experiential data to address these areas. Overall, the purpose of the personas is to build on the understanding about population experiences and perspectives, and to prompt the co-design of community care packages for this population.

The personas will also be presented to team members and advisors, who will be asked to draw on their collective experiences and share their perspectives. The research team has experience developing personas from both quantitative [12] and qualitative [48] data sources. In order to look at a range of risk, to investigate what prevention of hospital-admission might look like, and also prioritize those most at risk, the research team anticipates working with 6 risk profiles: three moderate-risk and three high-risk; however, this number may be adjusted depending on the risk profiles generated and recommendations from the research team and advisors. Half of the personas will be scenarios of people in hospitals, and the other half will be personas of people in the community.

Design researchers have noted that there is no perfect formula for determining the “right number” of personas to reflect the range of needs within a population (though it is recommended that less is more) [49]. For example, personas may not represent distinct types of people within a population but rather patterns reflected among a range of people. One option is to capture the average characteristics within the broader group, and the other is to capture the “opposite ends” of the range of group characteristics. Both options will be considered after generating the risk profiles and working with partners and advisors to select the best option.

To avoid the risk of losing focus on equity and diversity, the research team will work with participants and advisors to ensure these characteristics are not lost and are considered throughout the analysis. For example, the Delayed Hospital Discharge Patient and Caregiver Advisory Council will be consulted to seek their feedback on if the developing personas reflect community diversity and needs and in what areas inclusivity measures may be improved, whether for this or future studies. In either case, developing six personas across the moderate and high risk profiles may make it both feasible and representative of many of the populations’ needs [49].

### Focus groups and interviews

Multiple team members will conduct the focus groups and one-on-one interviews. The focus groups and interviews will be either online or in-person to accommodate participants’ preferences and availability. Older adults and caregivers, and healthcare providers and decision-makers will be separated into different focus groups for more in-depth discussions and to prompt openness and mitigate potential discomfort and power dynamics that may arise by having them in the same space.

During focus groups, the developed personas will be presented to participants for feedback including what resonates with them and what details need to be added. The guiding focus group questions will tap into various bio-psycho-social domains (e.g., “does the persona have someone they can count on when they need something?”, “describe the personas ability to engage in activities of daily living like eating and bathing”) [50]. Given the importance of understanding the experiences of people with cognitive impairment and their caregivers, the interviewers will probe for home and community care needs in the context of cognitive impairment, captured from participants who have the capacity to engage in the focus groups. Community and hospital-based service packages will then be co-designed with participants for the personas (drawing from health and social care resources).

Personas will be presented to participants for the first time during focus groups and interviews. Participants will be asked to: 1) discuss the persona by outlining whether it resonates and offer insights on what to add or remove, 2) identify challenges that might be experienced by the person depicted in the persona, and 3) outline their recommended services drawing on both existing and desired services/programs to illuminate assets and gaps. For the personas that are hospital-based, participants will be asked to share the types of services and programs that may help to mitigate harm and optimize functional outcomes of patients while they are in hospital. Services that are not currently available will be documented to support future planning and resource allocation. Barriers and facilitators to the implementation of the proposed care packages will be captured by asking questions such as, “what are the types of things that this persona would need support with?” and “what are the challenges that this persona may experience?” How components of the packages would shift based on sociodemographic characteristics (e.g., sex, gender, disability, ethnicity) as well as geographic service availability will be discussed. To co-design the care packages, the research team will prompt with questions such as, “which types of care providers might this persona need to interact with? What kind of support might they provide? How many hours per week do you think would be required of this provider to support the persona?” It is anticipated that each focus group will be up to 2 hours in duration. Participants will receive a note of thanks in tandem with their compensation.

### Qualitative data analysis

All focus group discussions will be audio-recorded and transcribed verbatim. The transcripts will be checked against the audio recording for accuracy, and identifiable information will be removed. The first round of focus group transcripts (persona development) will be analyzed to verify persona content, including areas of agreement and disagreement (to be revisited in subsequent focus groups and in consultation with the advisory council).

Second, inductive thematic analysis will be used following Braun and Clarke’s 6 steps to capture emergent themes from the focus group discussions [51]. The research team’s approach aligns with codebook thematic analysis, wherein a large codebook developed through analysis provides a degree of structure to theme development in order to meet “predetermined information needs … common in some areas of applied research” (p. 7) [52, 53]. For example, the current applied health services study requires that information is gained regarding how to better allocate services to older adults to avoid delayed hospital discharge. However, the research team does not use a fixed codebook. As analysis progresses, it is anticipated that the codebook will change in response to new insights gleaned from additional focus groups. The research team’s approach to analysis at times dips into reflexive thematic analysis through engagement with the ways in which the researcher has an impact on data collection and analysis, and view on codes and themes as a product of analysis conducted by a subjective researcher (and not necessarily latent in the data) [53].

Analysis will be conducted using Nvivo qualitative data analysis software [54]. The transcripts will also be reviewed to verify persona characteristics and care package components, drawing attention to areas of convergence and divergence as well as considerations for equity-deserving groups. Lincoln and Guba’s evaluative criteria for qualitative research [55] will be used to enhance the trustworthiness of the analysis (e.g., prolonged engagement with the data, peer debriefing, an audit trail of the analysis process, and reflexivity, which entails reflecting on how the research team’s varying roles and experiences impact data expectations). For each of the care package components, the research team will highlight which of the recommended services are already available (local assets) as well as what is required but not available (gaps).

### Knowledge mobilization forum

In order to follow up with participants, share findings, and further understand the feasibility of implementing the community and hospital care packages, a half-day, virtual multi-partner forum emulating World Café methods (which entails small group learning, sharing, and reflection) will be conducted [56]. At the beginning of the forum, the team will present the methodological approach (creation of risk profiles and personas) and focus group findings (personalization of the risk profiles from participant feedback) and the creation of care packages. Following that, participants will be broken up into virtual breakout rooms, each with a facilitator and a note-taker. Participants will be grouped by the role they most closely identify with (all older adults, all caregivers, all providers, and all decision-makers in their distinct groups) to address power differences and increase comfort in sharing ideas and reflections. In each breakout room, participants will be asked to share their perceived barriers and facilitators in implementing the care packages in practice. Following the virtual breakout room discussion, all participants will resume in the main virtual room for cross-group learning and sharing. A synthesis of findings and reflections will be presented back at the end of the virtual meeting and in a briefing note, circulated afterward.

## Discussion

### Potential risks and benefits

Participants may experience temporary emotional or psychological discomfort during the sessions as they may recall negative experiences or challenges they have encountered with their health, healthcare use, or provision. In the event participants experience distress or anxiety, they will be provided with a list of resources and contact information, including mental health support. Experiential evidence on delayed hospital discharge is crucial to understanding the impacts of delayed discharge. Participants’ contributions will serve as an important foundation for future investigations on other contexts across Canada.

### Limitations

There are limitations to the quantitative data available, which inform the development of the risk profiles and subsequent personas to be developed. Specifically, there is limited information on social determinants of health (e.g., housing, social support). To address this, a diverse sample of participants will be recruited, and probes during focus groups will aim at better understanding the unique perspectives and experiences of individuals who may not be adequately represented in the quantitative datasets. By asking about participant’s thoughts on what the persona would experience and need, and not necessarily asking participants about their direct personal lives, potential discomfort may be mitigated. The research team is also primarily English-speaking and can, therefore, only accommodate those with primary languages other than English through the use of an interpreter. While the research team is well-prepared and experienced in the use of interpreters and have done so in previous research, it is possible that this extra step may deter some non-English-speaking individuals from participating. There are also several limitations to consider in regard to ML predictions. For instance, the predictive models are developed using retrospective cohort data, with limited control over data collection, which can introduce selection bias. Additionally, the predictive models only focus on older delayed-discharge patients, therefore, their applicability to other populations is limited.

### Study impact

Delayed hospital discharge has been a critical care quality issue across Canada for decades. Health system leaders will be provided with an approach to 1) better match existing resources to the needs of older adults who are at risk of getting stuck in the hospital, 2) identify gaps in health and social care resources based on the characteristics, needs and preferences of older adults, their caregivers and providers; 3) combine two innovative methods to capture and assess population health, patient and provider experiences to support targeted health system planning for older adults; and 4) identify explicit direction and solutions for decision-makers in allocating services to avoid delayed hospital discharge.

## Supporting information

S1 Appendix COREQ checklist. (TIF)

S2 Appendix Persona worksheet. (DOCX)

S1 Checklist Human participants research checklist. (DOCX)

## References

[1] CBC News. Pressure Mounts for More Answers on Ford Government’s controversial long-term care bill. CBC News [Internet]. 2022 August 25 2022 August 26]. https://www.cbc.ca/news/canada/toronto/bill-7-ontario-long-term-care-hospitals-1.6561793.

[2] Canadian Institute for Health Information. Hospital stays in Canada [In: Hospital Stays in Canada—Series [Internet]. https://www.cihi.ca/en/hospital-stays-in-canada.

[3] Glasby J L R, Pryce K. All dressed up but nowhere to go? Delayed hospital discharges and older people. J Health Serv Res Policy. 2006;11(1):52–8. doi: 10.1258/135581906775094208 16378533

[4] Ontario. Premier’s council on improving healthcare and ending hallway medicine. Hallway health care: A system under strain: 1st interim report from the Premier’s council on improving healthcare and ending hallway medicine. 2019.

[5] Sutherland JM C R. Alternative level of care: Canada’s hospital beds, the evidence and options. Healthc Policy. 2013;1(9):26–34. 23968671 PMC3999549

[6] Walker D. Caring for aging population and addressing Alternate Level of Care. Report submitted to the Minister of Health and Long-Term Care. Toronto, ON; 2011.

[7] Canadian Institute for Health Information. Definitions and guidelines to support ALC designation in acute inpatient care. Ottawa, Canada: Canadian Institute for Health Information; 2016.

[8] Lyhne CN, Bjerrum M, Riis AH, Jørgensen MJ. Interventions to prevent potentially avoidable hospitalizations: a mixed methods systematic review. Front Public Health. 2022;10:898359. doi: 10.3389/fpubh.2022.898359 35899150 PMC9309492

[9] McGilton KS, Vellani S, Krassikova A, Robertson S, Irwin C, Cumal A, et al. Understanding transitional care programs for older adults who experience delayed discharge: a scoping review. BMC Geriatr. 2021;21:1–18. 33781222 10.1186/s12877-021-02099-9 PMC8008524

[10] Blum MR, Sallevelt BT, Spinewine A, O’Mahony D, Moutzouri E, Feller M, et al. Optimizing therapy to prevent avoidable hospital admissions in multimorbid older adults (OPERAM): cluster randomised controlled trial. BMJ. 2021;374. doi: 10.1136/bmj.n1585 34257088 PMC8276068

[11] Costa AP, Hirdes JP. Clinical characteristics and service needs of alternate-level-of-care patients waiting for long-term care in Ontario hospitals. Healthc Policy. 2010;6(1):32. 21804837 PMC2929891

[12] Kuluski K, Williams AP, Berta W, Laporte A. Home care or long-term care? Setting the balance of care in urban and rural Northwestern Ontario, Canada. Health Soc Care Community. 2012;20(4):438–48. doi: 10.1111/j.1365-2524.2012.01064.x 22582906

[13] Tucker S, Brand C, Wilberforce M, Challis D. The balance of care approach to health and social care planning: Lessons from a systematic literature review. Health Serv Manag Re. 2013;26(1):18–28. doi: 10.1177/0951484813481966 25594998

[14] Challis D, Hughes J. Frail old people at the margins of care: some recent research findings. Br J Psychiatry. 2002;180(2):126–30. doi: 10.1192/bjp.180.2.126 11823321

[15] Hughes J, Challis D. Frail older people–margins of care. Rev Clin Gerontol. 2004;14(2):155–64.

[16] Tucker S, Hughes J, Burns A, Challis D. The balance of care: reconfiguring services for older people with mental health problems. Aging Ment Health 2008;12(1):81–91. doi: 10.1080/13607860701366038 18297482

[17] Challis D, Tucker S, Wilberforce M, Brand C, Abendstern M, Stewart K, et al. National trends and local delivery in old age mental health services: towards an evidence base: a mixed-methodology study of the balance of care approach, community mental health teams and specialist mental health outreach to care homes. PGfAR. 2014;2(4):1–480. 27466646

[18] Clarkson P, Hughes J, Challis D. The potential impact of changes in public funding for residential and nursing-home care in the United Kingdom: the residential allowance. Ageing Soc. 2005;25(2):159–80.

[19] Williams AP, Challis D, Deber R, Watkins J, Kuluski K, Lum JM, et al. Balancing institutional and community-based care: why some older persons can age successfully at home while others require residential long-term care. Healthc Q (Toronto, Ont). 2009;12(2):95–105. doi: 10.12927/hcq.2009.3974 19369817

[20] Kuluski K, Peckham A, Williams AP. Setting the balance of care in Northwestern Ontario: opportunities and challenges for aging in place. Policy Report. 2015.

[21] Day S. A reflexive lens: exploring dilemmas of qualitative methodology through the concept of reflexivity. QSR. 2012;8(1):60–85.

[22] Leibing A, McLean A. “Learn to value your shadow!” An introduction to the margins of fieldwork. The shadow side of fieldwork: Exploring the blurred borders between ethnography and life 2007. p. 1–28.

[23] Leibing A, McLean A. When the borders of research and personal life become blurred: Thorny issues in conducting. The shadow side of fieldwork: Exploring the Blurred Borders Between Ethnography and Life. 2622007.

[24] Jacobson D, Mustafa N. Social identity map: a reflexivity tool for practicing explicit positionality in critical qualitative research. Int J Qual Methods. 2019;18:1609406919870075.

[25] Nixon SA. The coin model of privilege and critical allyship: implications for health. BMC Public Health. 2019;19(1):1637. doi: 10.1186/s12889-019-7884-9 31805907 PMC6896777

[26] Patel A, Regier K, Wilson K, Ghassemi E, Dean J. Beyond the cosmopolis: Sustaining hyper-diversity in the suburbs of Peel Region, Ontario. Urban Plan. 2018;3(4):38–49.

[27] Simcoe Muskoka District Health Unit. Population [cited 2024 May 28]. In: HealthSTATS Simcoe Muskoka [Internet]. https://www.simcoemuskokahealth.org/Health-Stats/HealthStatsHome/PopulationDemographics/Population.

[28] Unit SMDH. Quality of health [cited 2024 May 28]. In: HealthSTATS Simcoe Muskoka [Internet]. https://www.simcoemuskokahealth.org/Health-Stats/HealthStatsHome/PopulationDemographics/QualityofHealth.

[29] University of Toronto. Humber River Hospital 2023 [cited 2023 February 21]. https://dfcm.utoronto.ca/humber-river-hospital#:~:text=Facility%20(Wilson)%3A-,Home%20to%20over%204%2C000%20employees%2C%20700%20physicians%20and%20500%20volunteers,and%2030%2C000%20outpatient%20surgeries%20annually.

[30] Peel Region. Peel region data portal [cited 2024 April 30]. In: Data Portal [Internet]. https://data.peelregion.ca/.

[31] Ghazalbash S, Zargoush M, Mowbray F, Papaioannou A. Examining the predictability and prognostication of multimorbidity among older Delayed-Discharge Patients: a machine learning analytics. Int J Med Inform. 2021;156:104597. doi: 10.1016/j.ijmedinf.2021.104597 34619571

[32] Lundberg SM, Lee S-I. A unified approach to interpreting model predictions. Adv Neural Inf Process Syst. 2017;30.

[33] Staniak M, Biecek P. Explanations of model predictions with live and breakDown packages. R J. 2018.

[34] Chen T, Guestrin C, editors. XGBoost: A Scalable Tree Boosting System. Conference on Knowledge Discovery and Data Mining (KDD); 2016; San Francisco, CA, USA.

[35] Ravaut M, Sadeghi H, Leung K, Volkovs M, Kornas K, Harish V, et al. Predicting adverse outcomes due to diabetes complications with machine learning using administrative health data. NPJ digital medicine. 2021;4(1):1–12. 33580109 10.1038/s41746-021-00394-8 PMC7881135

[36] Ravaut M, Harish V, Sadeghi H, Leung KK, Volkovs M, Kornas K, et al. Development and validation of a machine learning model using administrative health data to predict onset of type 2 diabetes. JAMA network open. 2021;4(5):e2111315–e. doi: 10.1001/jamanetworkopen.2021.11315 34032855 PMC8150694

[37] Gutierrez JM, Volkovs M, Poutanen T, Watson T, Rosella LC. Risk stratification for COVID-19 hospitalization: a multivariable model based on gradient-boosting decision trees. CMAJ Open. 2021;9(4):E1223–E31. doi: 10.9778/cmajo.20210036 34933880 PMC8695533

[38] Eun Yi S, Harish V, Gutierrez J, Ravaut M, Kornas K, Watson T, et al. Predicting hospitalizations related to ambulatory care sensitive conditions with machine learning for population health planning: derivation and validation cohort study. BMJ Open. 2021;Pre Print. 10.1136/bmjopen-2021-051403 PMC8977821 35365510

[39] Lundberg S, Erion G, Chen H, DeGrave A, Prutkin J, Nair B, et al. From local explanations to global understanding with explainable AI for trees. Nature machine intelligence. 2020;2(1):56–67. doi: 10.1038/s42256-019-0138-9 32607472 PMC7326367

[40] Mowbray F, Zargoush M, Jones A, de Wit K, Costa A. Predicting hospital admission for older emergency department patients: Insights from machine learning. Int J Med Inform. 2020;140:104163. doi: 10.1016/j.ijmedinf.2020.104163 32474393

[41] Zargoush M, Alemi F, Esposito Vinzi V, Vang J, Kheirbek R. A psychological approach to learning causal networks. Health Care Manag Sci. 2014;17(2):194–201. doi: 10.1007/s10729-013-9250-2 24048957

[42] Tong A, Sainsbury P, Craig J. Consolidated criteria for reporting qualitative research (COREQ): a 32-item checklist for interviews and focus groups. Int J Qual Health. 2007;19(6):349–57. doi: 10.1093/intqhc/mzm042 17872937

[43] Harrison JD, Auerbach AD, Anderson W, Fagan M, Carnie M, Hanson C, et al. Patient stakeholder engagement in research: a narrative review to describe foundational principles and best practice activities. Health Expect. 2019;22(3):307–16. doi: 10.1111/hex.12873 30761699 PMC6543160

[44] Regenold N, Vindrola-Padros C. Gender matters: A gender analysis of healthcare workers’ experiences during the first COVID-19 pandemic peak in England. Soc Sci. 2021;10(2):43.

[45] Malterud K, Siersma VD, Guassora AD. Sample size in qualitative interview studies: guided by information power. Qual Health Res. 2016;26(13):1753–60. doi: 10.1177/1049732315617444 26613970

[46] Bak K, Moody L, Wheeler SM, Gilbert J. Patient and staff engagement in health system improvement: a qualitative evaluation of the Experience-Based Co-design Approach in Canada. Healthc Q(Toronto, Ont). 2018;21(2):24–9. doi: 10.12927/hcq.2018.25626 30474588

[47] Kuluski K, Ho JW, Cadel L, Shearkhani S, Levy C, Marcinow M, et al. An alternate level of care plan: Co-designing components of an intervention with patients, caregivers and providers to address delayed hospital discharge challenges. Health Expect. 2020;23(5):1155–65. doi: 10.1111/hex.13094 32602628 PMC7696114

[48] Kuluski K, Ho JW, Hans PK, Nelson ML. Community care for people with complex care needs: bridging the gap between health and social care. Int J Integr Care. 2017;17(4). 10.5334/ijic.2944 PMC5624113 28970760

[49] M G. How many personas do I need? 2020 [cited 2022 March 1]. In: Medium [Internet]. https://mattgramcko.medium.com/how-many-personas-do-i-need-5f69248c1e99.

[50] Schaink AK, Kuluski K, Lyons RF, Fortin M, Jadad AR, Upshur R, et al. A scoping review and thematic classification of patient complexity: offering a unifying framework. J Comorb. 2012;2(1):1–9. doi: 10.15256/joc.2012.2.15 29090137 PMC5556402

[51] Braun V, Clarke V. Using thematic analysis in psychology. Qual Res Psychol. 2006;3(2):77–101.

[52] Braun V, Clarke V. Toward good practice in thematic analysis: Avoiding common problems and be (com) ing a knowing researcher. Int J Transgender Health. 2023;24(1):1–6. 10.1080/26895269.2022.2129597 PMC9879167 36713144

[53] Braun V, Clarke V. Conceptual and design thinking for thematic analysis. Qual Psychol. 2022;9(1):3.

[54] Lumivero. NVivo 14—Leading qualitative data analysis software with AI solution: Lumivero; 2024 [cited 2024 April 16]. In: Lumivero [Internet]. https://lumivero.com/products/nvivo/.

[55] Lincoln YS, Guba EG. Naturalistic inquiry: sage; 1985.

[56] Löhr K, Weinhardt M, Sieber S. The “World Café” as a participatory method for collecting qualitative data. Int J Qual Methods. 2020;19:1609406920916976.

