# Supplementary figures and images for: Setting the balance of care for older adults at risk of hospitalization and delayed discharge: A mixed-methods research protocol
Source: PLoS One. 2024 Dec 17;19(12):e0315918. doi: 10.1371/journal.pone.0315918 (PMC11651538; doi:10.1371/journal.pone.0315918)

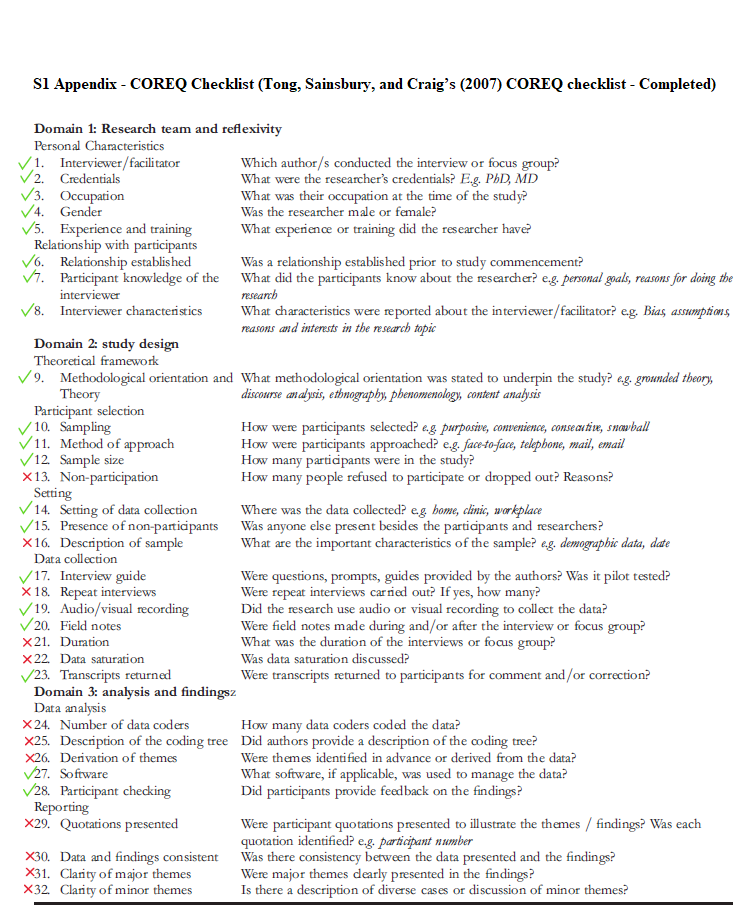

Supplement: S1 Appendix — (TIF) [file pone.0315918.s001.tif]
