# Supplementary material for: Setting the balance of care for older adults at risk of hospitalization and delayed discharge: A mixed-methods research protocol
Source: PLoS One. 2024 Dec 17;19(12):e0315918. doi: 10.1371/journal.pone.0315918 (PMC11651538; doi:10.1371/journal.pone.0315918)
Supplement: S2 Appendix — (DOCX) [file pone.0315918.s002.docx]

**S2 Appendix - Persona Development Worksheet**

| **My name is:**  ___________________ | 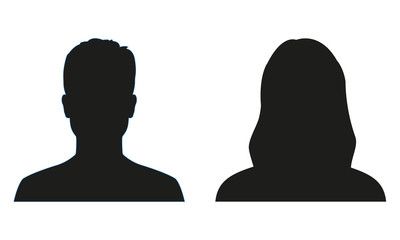  **I am _____ years old**  **My gender identity is:** Man Woman Transgender Non-binary Gender fluid Gender non conforming or gender queer Two Spirit Questioning or Unsure |
| --- | --- |
| **Marital Status:** | |
| **Employment Status**  **Full time**  **Part time/casual**  **Retired**  **Not employed at this time** | |
| **I was born in:** | |
| **I identify as (check all that apply):**   - **First Nation** - **Inuit** - **Métis** - **Indigenous/Aboriginal** - **Arab** - **Black (North American, Caribbean, African, etc.)** - **Chinese** - **Filipino** - **Japanese** - **Korean** - **Latin American** - **South Asian (East Indian, Pakistani, Sri Lankan, etc.)** - **Southeast Asian (Vietnamese, Cambodian, Malaysian, Laotian, etc.)** - **West Asian (Iranian, Afghan, etc.)** - **White (North American, European, etc.)**   **Other (please specify):** | |

| **The primary language that I speak is:** |
| --- |
| **I have enough money to meet my daily living needs**  **Yes No** |
| **Living arrangements:**  **on own with others**  **homeless** |
| **Description of current residence (housing, if applicable):** |
| **Description of my neighborhood (proximity to amenities, walkability, safety):** |
| **I have someone I can count on when I need something (such as a friend or family member)**  **Yes No** |
| **This person provides** [add description of support]  **How many hours? ___**  **Per week**  **Per day** |
| **This person lives in the same city as me**  **Yes**  **No** |
| **Description of ability to do activities of daily living (eating, bathing, toileting, etc.):** |
| **Description of ability to do instrumental activities of daily living (making meals, housekeeping, paying bills, driving to appointments):** |
| **How do you categorize your ability to make decisions about your daily care needs?**  **Independent**  **Needing supervision**  **Needing some help**  **Needing significant help**  **Dependent on someone else** |
| **Is faith (religion, beliefs) important to you?**  **Yes**  **No**  **Add additional content as needed about the influence of faith and regular practices:** |
| **Do you engage in specific cultural practices?**  **Yes**  **No**  **Add additional content as needed about cultural influence and practices:** |
| **I currently interact with the following types of care providers:** |
| **I have a Family Doctor I can visit when I need medical attention**  **Yes No**  **Add additional content as needed about family doctor:** |
| **I prefer to receive services in my primary language**  Yes No |
| **The types of things I need support with are:** |
| **The types of challenges I experience are:** |
| **Other important details about me**: |
